# Stringent Response-Mediated Control of GTP Homeostasis Is Required for Long-Term Viability of Staphylococcus aureus

**DOI:** 10.1128/spectrum.00447-23

**Published:** 2023-03-06

**Authors:** Laura Carrilero, Lucy Urwin, Ezra Ward, Naznin R. Choudhury, Ian R. Monk, Claire E. Turner, Timothy P. Stinear, Rebecca M. Corrigan

**Affiliations:** a The Florey Institute, School of Biosciences, University of Sheffield, Sheffield, United Kingdom; b Department of Microbiology and Immunology, Doherty Institute for Infection and Immunity, University of Melbourne, Melbourne, Victoria, Australia; University of Florida College of Dentistry

**Keywords:** (p)ppGpp, GTP, *Staphylococcus aureus*, stringent response

## Abstract

Staphylococcus aureus is an opportunistic bacterial pathogen that often results in difficult-to-treat infections. One mechanism used by S. aureus to enhance survival during infection is the stringent response. This is a stress survival pathway that utilizes the nucleotides (p)ppGpp to reallocate bacterial resources, shutting down growth until conditions improve. Small colony variants (SCVs) of S. aureus are frequently associated with chronic infections, and this phenotype has previously been linked to a hyperactive stringent response. Here, we examine the role of (p)ppGpp in the long-term survival of S. aureus under nutrient-restricted conditions. When starved, a (p)ppGpp-null S. aureus mutant strain ((p)ppGpp^0^) initially had decreased viability. However, after 3 days we observed the presence and dominance of a population of small colonies. Similar to SCVs, these small colony isolates (p^0^-SCIs) had reduced growth but remained hemolytic and sensitive to gentamicin, phenotypes that have been tied to SCVs previously. Genomic analysis of the p^0^-SCIs revealed mutations arising within *gmk*, encoding an enzyme in the GTP synthesis pathway. We show that a (p)ppGpp^0^ strain has elevated levels of GTP, and that the mutations in the p^0^-SCIs all lower Gmk enzyme activity and consequently cellular GTP levels. We further show that in the absence of (p)ppGpp, cell viability can be rescued using the GuaA inhibitor decoyinine, which artificially lowers the intracellular GTP concentration. Our study highlights the role of (p)ppGpp in GTP homeostasis and underscores the importance of nucleotide signaling for long-term survival of S. aureus in nutrient-limiting conditions, such as those encountered during infections.

**IMPORTANCE** Staphylococcus aureus is a human pathogen that upon invasion of a host encounters stresses, such as nutritional restriction. The bacteria respond by switching on a signaling cascade controlled by the nucleotides (p)ppGpp. These nucleotides function to shut down bacterial growth until conditions improve. Therefore, (p)ppGpp are important for bacterial survival and have been implicated in promoting chronic infections. Here, we investigate the importance of (p)ppGpp for long-term survival of bacteria in nutrient-limiting conditions similar to those in a human host. We discovered that in the absence of (p)ppGpp, bacterial viability decreases due to dysregulation of GTP homeostasis. However, the (p)ppGpp-null bacteria were able to compensate by introducing mutations in the GTP synthesis pathway that led to a reduction in GTP build-up and a rescue of viability. This study therefore highlights the importance of (p)ppGpp for the regulation of GTP levels and for long-term survival of S. aureus in restricted environments.

## INTRODUCTION

Pathogenic bacteria, including Staphylococcus aureus, have complex gene regulatory mechanisms to promote survival in changing environments such as the human host. The stringent response is a global stress adaptation pathway used by bacteria to deal with environmental challenges, including nutrient starvation and pH and temperature stresses (1, 2). This response is coordinated by the rapid synthesis of the nucleotides guanosine tetra- and pentaphosphate, collectively termed (p)ppGpp, which control a cellular switch resulting in slowed growth and stress adaptation (1, 3). Numerous reports to date also highlight a role for the stringent response in S. aureus virulence and its contribution to persistent and chronic infections (4 to 9).

In S. aureus, (p)ppGpp is synthesized by three synthetase enzymes, Rel, RelP, and RelQ (9). While RelP and RelQ respond to stresses like cell-wall-targeting antibiotics and alterations in pH (10), Rel synthesizes (p)ppGpp during amino acid depletion, leading to large transcriptional changes, including the downregulation of protein synthesis machinery and upregulation of amino acid transport and metabolism genes (4). As S. aureus is a conditional auxotroph for a number of amino acids (11, 12), this regulation of amino acid uptake is essential for survival in starved conditions (9). In proteobacteria, (p)ppGpp modulates changes in transcription of these genes by binding to two sites around the β′ subunit of the RNA polymerase, leading to altered expression of roughly a third of the genome (13). However, in S. aureus, and other Firmicutes, these interactions with the RNAP core enzyme do not occur (14 to 16). Instead, (p)ppGpp mediates changes by inhibiting a number of proteins, including those involved in DNA replication and ribosome assembly and translation, as well as by active depletion of GTP (1, 15, 17, 18).

Upon induction of (p)ppGpp synthesis, GTP levels drop due to its use as a substrate for pppGpp synthesis. In addition, (p)ppGpp also inhibits a number of GTP biosynthetic enzymes, including the IMP dehydrogenase GuaB and the guanylate kinase Gmk, both involved in the *de novo* GTP synthesis pathway (1, 19). This reduction in cellular GTP reduces transcription from promoters that use GTP as their initiating nucleotide, which includes promoters for rRNA synthesis, profoundly impacting cellular physiology (14, 15, 20, 21). Additionally, in the Firmicutes, the decrease in GTP level allows for the derepression of genes under the control of the CodY transcriptional repressor. CodY is a global regulator, where it functions to repress genes involved in amino acid biosynthesis, amino acid uptake, and some virulence genes (22). Studies using (p)ppGpp-null strains of Bacillus subtilis have observed that subjecting strains lacking (p)ppGpp to amino acid-depleted media for a 10 min shock resulted in cell death. This was attributed to elevated GTP concentrations, making (p)ppGpp-mediated control of GTP levels crucial for cell viability (16, 19, 23).

Persistent and recurring S. aureus infections are often associated with a small colony variant (SCV) phenotype on solid media (24, 25). Classical SCV phenotypes from clinical strains include colonies that are nonhemolytic, nonpigmented, slower growing, resistant to antibiotics, and often auxotrophic for menadione, hemin, or thymidine (24, 25). Additionally, these SCV strains can have an unstable small colony phenotype that can revert to normal-sized colonies (24, 26, 27). In some SCVs, an activated stringent response has been reported. A clinical S. aureus SCV strain isolated from a patient with a persistent and recurrent infection had a point mutation resulting in permanent activation of the stringent response (5). This contributed to slowed growth and reduced virulence, and highlighted the survival advantage that the stringent response provides during infection.

In this work, we sought to determine the requirement of (p)ppGpp for long-term survival of S. aureus, with the aim of providing further insights into its importance for recurrent infection. We observed that the absence of (p)ppGpp resulted in an S. aureus strain with increased GTP levels and an initial viability defect when cells are grown in nutrient-restricted media. However, over time, the bacterial culture was subsequently dominated by a population of smaller-sized colonies, distinct from traditional SCVs, that restored viability counts to wild-type levels. Whole-genome sequencing of the smaller colonies revealed the presence of mutations arising within *gmk*, encoding an enzyme of the GTP synthesis pathway. The overall effect of this was lowered enzyme activity and GTP levels in these cells, which allowed for the survival of strains lacking the stringent response in nutrient-limited media. Our data suggest that, similar to B. subtilis, (p)ppGpp is an important regulator of GTP homeostasis in S. aureus but that this regulation can be otherwise compensated for by suppressor mutations in the GTP synthesis pathway.

## RESULTS

### (p)ppGpp^0^ S. aureus form smaller colonies when starved.

(p)ppGpp, while not essential when strains are grown in rich laboratory media, is required for the growth of S. aureus in nutrient-restricted media (2). To examine the requirement of (p)ppGpp for long-term survival in media that is more physiologically relevant, we first constructed a (p)ppGpp-null mutant in the methicillin-resistant USA300 S. aureus strain JE2 (JE2 Δ*relQPA*) by introducing silent in-frame deletions in the three (p)ppGpp synthetases: *rel*, *relP*, and *relQ*. The triple mutant, termed (p)ppGpp^0^, was unable to produce (p)ppGpp after a 30-min shock with mupirocin (Fig. S1A in the supplemental material), a known inducer of (p)ppGpp synthesis (28). A concurrent increase in GTP levels was also observed (Fig. S1A), as has been noted for B. subtilis (p)ppGpp^0^ strains (19). (p)ppGpp^0^ also displayed characteristic growth defects when grown in amino-acid-starved conditions (Fig. S1B and C), with whole-genome sequencing verifying the absence of secondary mutations in the strain.

**FIG 1:**
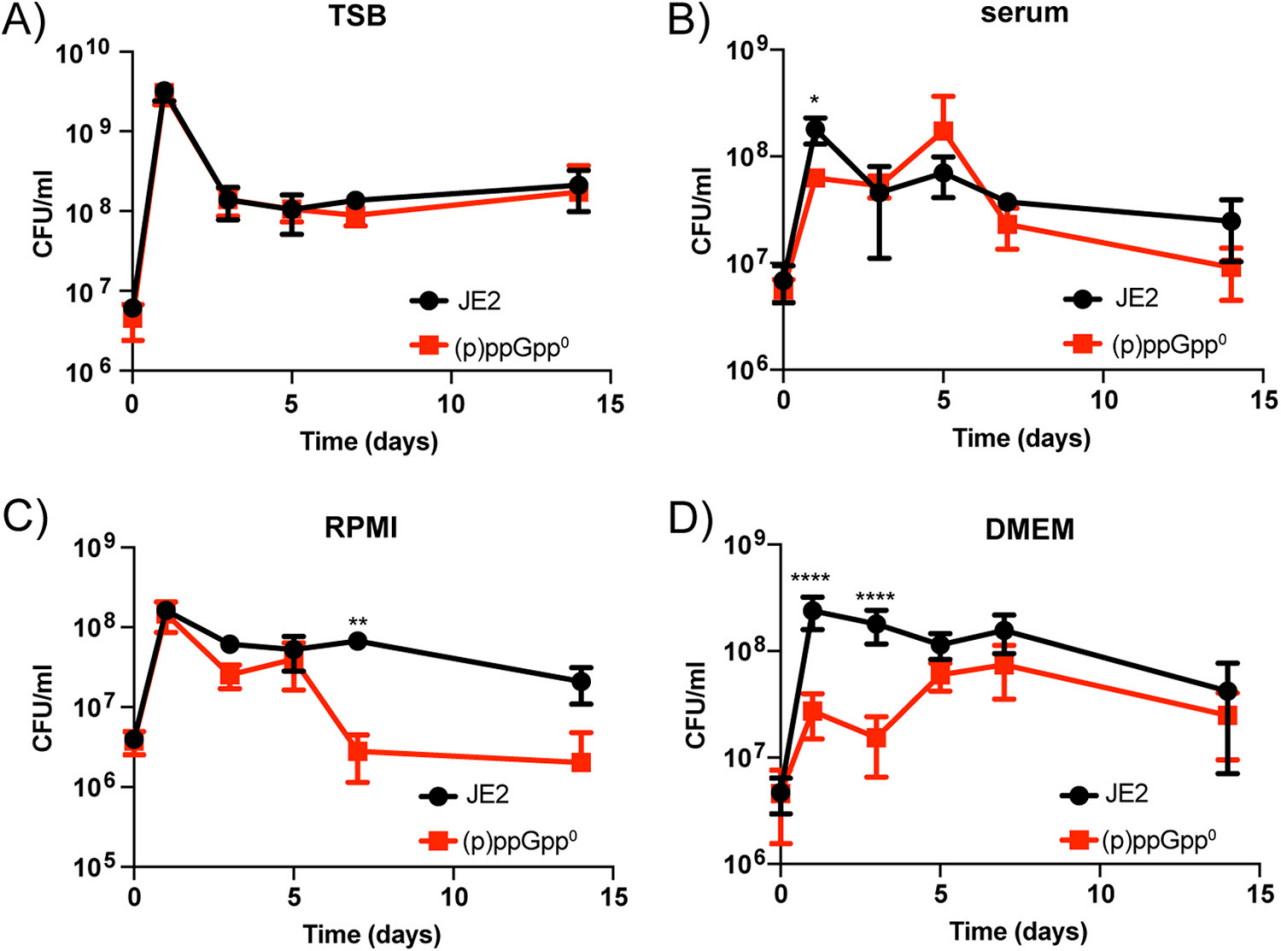
Survival of wild-type and (p)ppGpp^0^ strains in nutrient-rich and nutrient-restricted media. Changes in viable count (CFU/mL) were measured over 14 days for JE2 (black) and (p)ppGpp^0^ (red) in (A) TSB, (B) human serum, (C) RPMI, and (D) DMEM with l-glutamine and FBS. Survival curves were carried out in triplicate, with error bars representing standard deviation. Data were analyzed by two-way ANOVA with Šidák’s multiple-comparison test, *, *P* ≤ 0.05; **, *P* ≤ 0.01; ****, *P* ≤ 0.0001.

We then compared the ability of both wild-type JE2 and (p)ppGpp^0^ strains to survive long-term starvation in nutrient-rich tryptic soy broth (TSB) and three nutrient-deplete conditions: human serum, RPMI (iron-depleted cell culture media), and DMEM (minimal essential cell culture media) supplemented with glutamine and fetal bovine serum (FBS) over 14 days (Fig. 1). Following an initial increase in CFU at day one in TSB, both the wild-type and the (p)ppGpp^0^ strains ceased growing with a log drop in viable cells by day three (Fig. 1A). The number of cells for both strains then remained constant over the remainder of the 14-day period, demonstrating that in rich media both strains are capable of long-term survival. In human serum, there were only minor differences in viable cell counts between the strains over the 14 days (Fig. 1B); however, in RPMI, a two-log drop in CFU was observed for the (p)ppGpp^0^ mutant after 7 days (Fig. 1C). RPMI is a cell culture medium originally formulated to grow human lymphocytes, and is rich in amino acids but restricted in iron content. (p)ppGpp has previously been reported as important for maintaining iron homeostasis in S. aureus (29, 30), in line with what was observed here.

**FIG 2:**
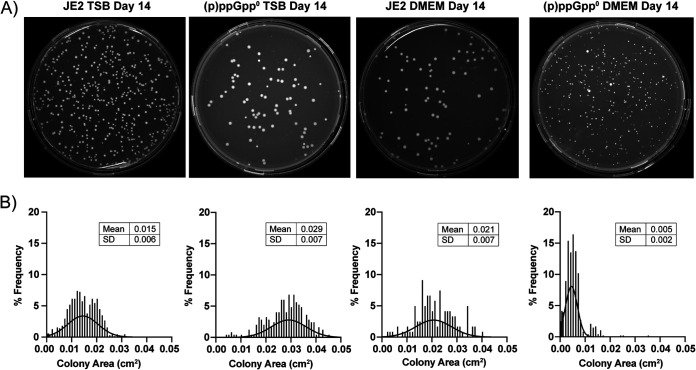
(p)ppGpp^0^ S. aureus form smaller colonies when starved. (A) Representative agar plates displaying JE2 and (p)ppGpp^0^ colony morphology after 14 days culture in TSB or DMEM. (B) Histograms showing the % frequency of colonies for a given size (in cm^2^) for JE2 and (p)ppGpp^0^ populations after 14 days in TSB or DMEM. Experiments were performed in triplicate.

DMEM is another cell culture medium that can support the growth of many cell types, but it is lacking in a number of amino acids, including asparagine, aspartic acid, and proline. In this medium, the (p)ppGpp^0^ strain displayed a significant decrease in CFU compared to the wild-type up until day five, when the colony numbers recovered to similar levels (Fig. 1D). By observing the colony morphology over time, it was apparent that colonies with a smaller phenotype began to appear for the (p)ppGpp^0^ strain in DMEM from day three, while the wild-type colony size was unaffected. By day 14, the (p)ppGpp^0^ population was dominated by the smaller colonies (Fig. 2A), with the mean colony size reducing from 0.021 ± 0.007 cm^2^ for the wild-type to 0.005 ± 0.002 cm^2^ for the mutant when grown in DMEM (Fig. 2B). To uncover whether the initial survival defect and the emergence of the smaller colonies observed when grown in DMEM were due to one specific (p)ppGpp synthetase or to the presence of the (p)ppGpp alarmones in general, strains with single deletions in *relP* and *relQ* and a double *relP relQ* mutant were compared to the triple mutant (Fig. S2A). As deleting *rel* individually in the presence of RelP and RelQ is not possible due to toxic accumulation of (p)ppGpp, a strain with a nonfunctional *rel* synthetase domain was also compared to the triple mutant to specifically examine the contribution of Rel (Fig. S2B). We also compared a mutant in *codY*, the transcriptional repressor interlinked with (p)ppGpp signaling (Fig. S2A). No survival defects or small colonies were observed in these strains (Fig. S2), suggesting that the presence of (p)ppGpp itself is required to survive in nutrient-limited DMEM medium.

### (p)ppGpp^0^ small colonies are phenotypically distinct from traditional SCVs.

**FIG 3:**
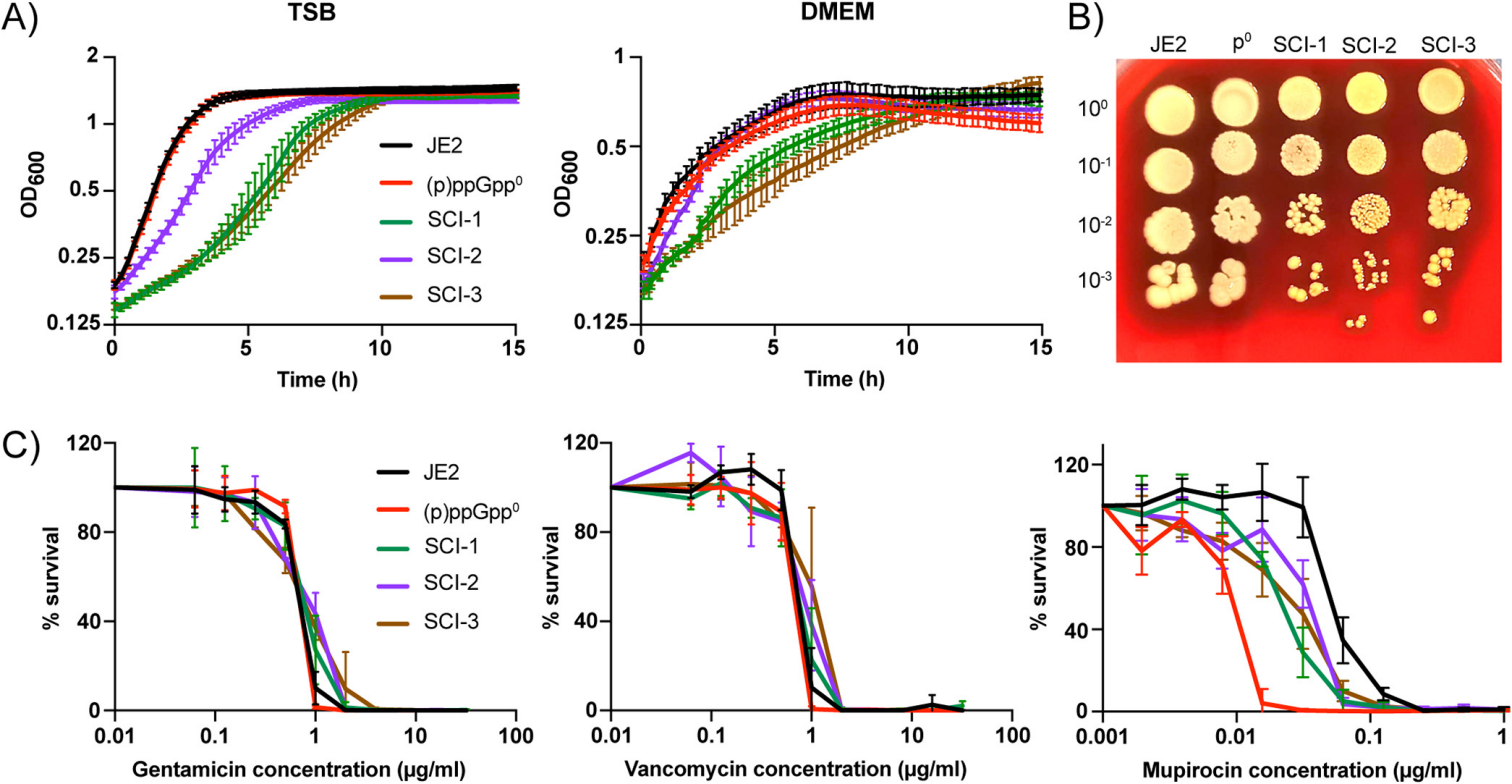
Phenotypic analysis of S. aureus (p)ppGpp^0^ small colony isolates. (A) Growth curves of JE2, (p)ppGpp^0^, p^0^-SCI-1, p^0^-SCI-2, and p^0^-SCI-3 in TSB and DMEM. Overnight cultures were diluted to an OD_600_ of 0.05 and grown for 15 h. Graphs show the mean OD_600_ of three replicates, with the standard deviation shown. (B) Serial dilutions of JE2, (p)ppGpp^0^, p^0^-SCI-1, p^0^-SCI-2, and p^0^-SCI-3 were spotted onto TSA plates containing 5% sheep’s blood and incubated at 37°C for 48 h. (C) MIC values of JE2, (p)ppGpp^0^, p^0^-SCI-1, p^0^-SCI-2, and p^0^-SCI-3 for gentamicin, vancomycin, and mupirocin were determined after overnight growth of each strain in the presence of doubling dilutions of each antibiotic. OD_600_ readings were determined after 24 h growth and plotted as % growth compared with the growth in the absence of antibiotic. Plots show the mean OD_600_ of three replicates and their corresponding standard deviation.

S. aureus SCVs can arise within a wild-type population when the cells are placed under stress, including sublethal concentrations of hydrogen peroxide, antibiotic stress, osmotic stress, low temperature, and acidic pH (31, 32). One typically reported characteristic of SCVs is their slow growth in comparison to wild-type strains. Often this slow growth phenotype is a result of a defect in cellular respiration (33), with mutations in genes involved in the electron transport chain (34, 35). The growth of three S. aureus (p)ppGpp^0^ small colony isolates (SCIs) from three separate biological experiments (p^0^-SCI-1, p^0^-SCI-2, and p^0^-SCI-3) were monitored for 15 h in both TSB and DMEM (Fig. 3A, Table S1). In TSB, the wild-type and the (p)ppGpp^0^ parental strain had a similar growth profile, while all three SCIs exhibited a growth defect. The growth defect of p^0^-SCI-2, however, was less pronounced than that of p^0^-SCI-1 and p^0^-SCI-3. In DMEM, all strains exhibited decreased growth compared to TSB-grown cells, with p^0^-SCI-1 and p^0^-SCI-3 growing less well than JE2 and (p)ppGpp^0^, as in rich media. Most notably, the growth rate of p^0^-SCI-2 was recovered in the nutrient-poor DMEM media, reaching stationary phase at a similar time as the wild-type and the (p)ppGpp^0^ strain, suggesting that this strain has an adaption to nutrient-poor media that increases fitness and which differs to p^0^-SCI-1 and p^0^-SCI-3.

Typical S. aureus SCVs have been described as having decreased hemolytic activity (36). As such, we investigated the hemolytic activity of the three (p)ppGpp^0^ SCIs by plating 10-fold serial dilutions of the strains onto blood-agar plates; however, lytic activity was apparent for all strains (Fig. 3B). S. aureus SCVs are also commonly associated with recurring, antibiotic-resistant infections that can be difficult to treat (37). Here, the MICs of the three (p)ppGpp^0^ SCIs to mupirocin, vancomycin, and gentamicin were determined (Fig. 3C). Gentamicin and vancomycin resistance has been associated with SCVs (37, 38), while mupirocin is an antibiotic that inhibits the isoleucyl tRNA-synthetase and induces amino acid starvation, activating (p)ppGpp synthesis in a wild-type strain (28). The MIC of mupirocin for all three SCIs was determined to be 0.0625 μg/mL, a 4-fold increase compared to their (p)ppGpp^0^ parent strain and only a 2-fold decrease compared to the wild-type (Fig. 3C). However, the MIC of the SCIs for vancomycin and gentamicin showed no significant difference. These data suggest that in the absence of (p)ppGpp, when strains should be more sensitive to amino acid starvation, mutations have arisen in the SCIs to improve survival.

### Mutations in the *gmk* gene are present in all three (p)ppGpp^0^ small colony isolates.

**FIG 4:**
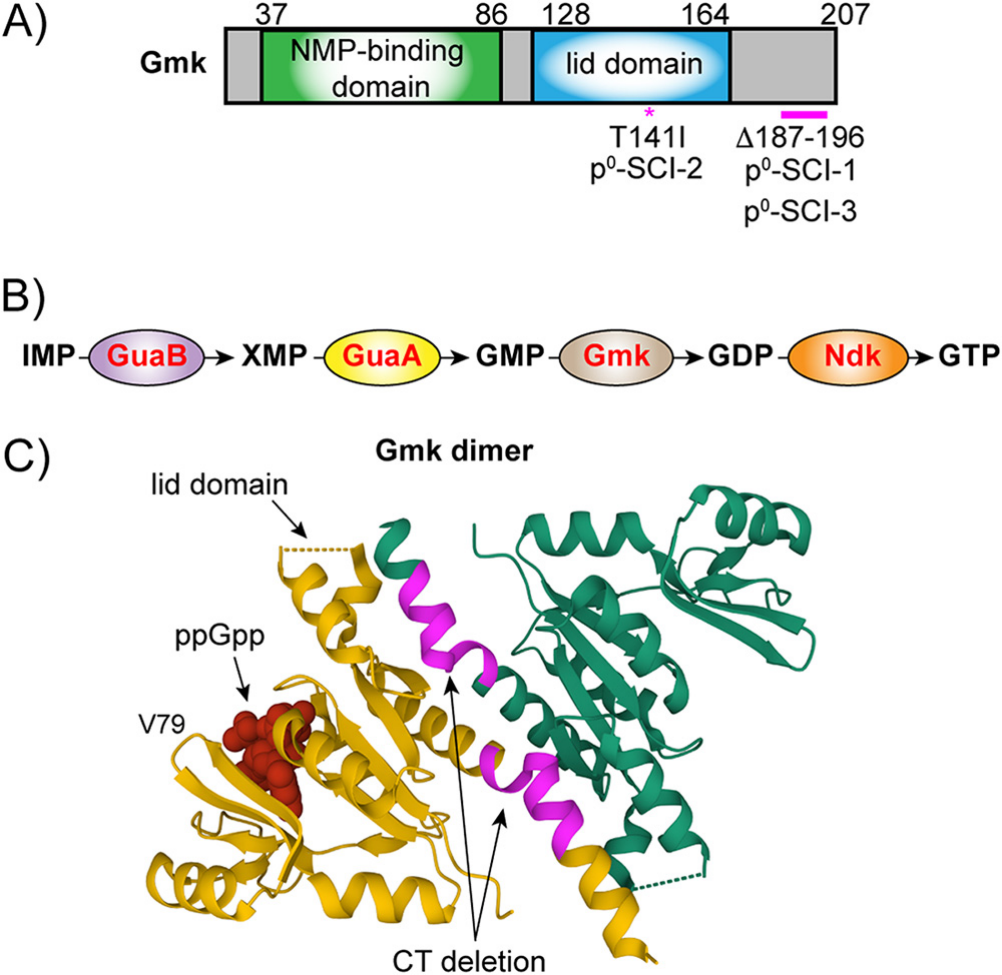
S. aureus (p)ppGpp^0^ small colony isolates contain mutations within Gmk. (A) Schematic representation of the mutations found in Gmk. Gmk contains two domains, the nucleoside monophosphate (NMP)-binding domain in green and the lid domain in blue. The sizes of each domain are indicated by numbering. Deletions found in p^0^-SCI-1 and p^0^-SCI-3 are represented by a pink line. *, an amino acid change in the lid domain in p^0^-SCI-2. (B) GTP synthesis pathway. Gmk (brown) catalyzes the conversion of GMP to GDP, a reaction that is inhibited by (p)ppGpp. (C) The crystal structure of Gmk from S. aureus as a dimer (PDB: 4QRH), with one monomer in yellow bound to ppGpp (red). The lid domain region containing the T141I mutation that is not resolved is indicated. Regions from amino acids 187 to 196 at the dimer interface that are lacking in both p^0^-SCI-1 and p^0^-SCI-3 are highlighted in pink.

To ascertain whether mutations had arisen in the three (p)ppGpp^0^ SCIs that were now allowing the strains to survive in DMEM, whole-genome sequencing was performed. Both p^0^-SCI-1 and p^0^-SCI-3 contained identical in-frame deletions of a sequence encoding 10 amino acids toward the C terminus of the protein Gmk (Fig. 4A). Both strains also had either a single nucleotide polymorphism (SNP) in the promoter region (p^0^-SCI-1) or a SNP introducing a frameshift mutation (p^0^-SCI-3) in SAUSA300_RS12245, encoding a small 56 amino acid protein of unknown function. However, sanger sequencing of both *gmk* and SAUSA300_RS12245 from the isolates at day 5, from each biological replicate, showed that only mutations in *gmk* were present. This rules out the direct involvement of SAUSA300_RS12245 in forming the small colony phenotype that arose on day three, though it may have been introduced later to aid growth. Isolate p^0^-SCI-2, on the other hand, had only one unique SNP compared to the parental (p)ppGpp^0^, a transition mutation yielding Gmk T141I (Fig. 4A).

Gmk is a guanylate kinase responsible for the conversion of GMP to GDP, which is then converted to GTP by the nucleoside diphosphate kinase Ndk (Fig. 4B). (p)ppGpp is a known binder of Gmk, where it can competitively inhibit guanylate kinase activity, and thus, when the stringent response is triggered in wild-type cells, GTP levels decrease as less GDP is present for conversion (16, 19, 39). This decrease in GTP then relieves repression by the transcriptional repressor CodY, increasing transcription of amino acid biosynthesis genes, allowing cells to survive amino acid starvation (22, 40, 41).

When active, the S. aureus Gmk is dimeric (42), with the C terminus of the protein crucial for dimer formation (Fig. 4C). When unbound by nucleotides, this protein is in an open conformation. The binding of ATP and GMP causes a conformational change to the closed state, whereby the lid domain moves and forms bonds with the ATP. The closure of the lid allows the protein to form a catalytically active state, forming GDP from ATP and GMP. Mapping the SCI mutations onto the structure of Gmk revealed that the 10 amino-acid C-terminal deletions within p^0^-SCI-1 and p^0^-SCI-3 are within helix 8, which is crucial for dimer formation, while the T141I mutation found in p^0^-SCI-2 is within the lid domain. Both of these regions are important for enzyme function (42) and suggest that these Gmk variants might have altered guanylate kinase activity.

### (p)ppGpp^0^ small colony isolates have reduced GTP levels.

**FIG 5:**
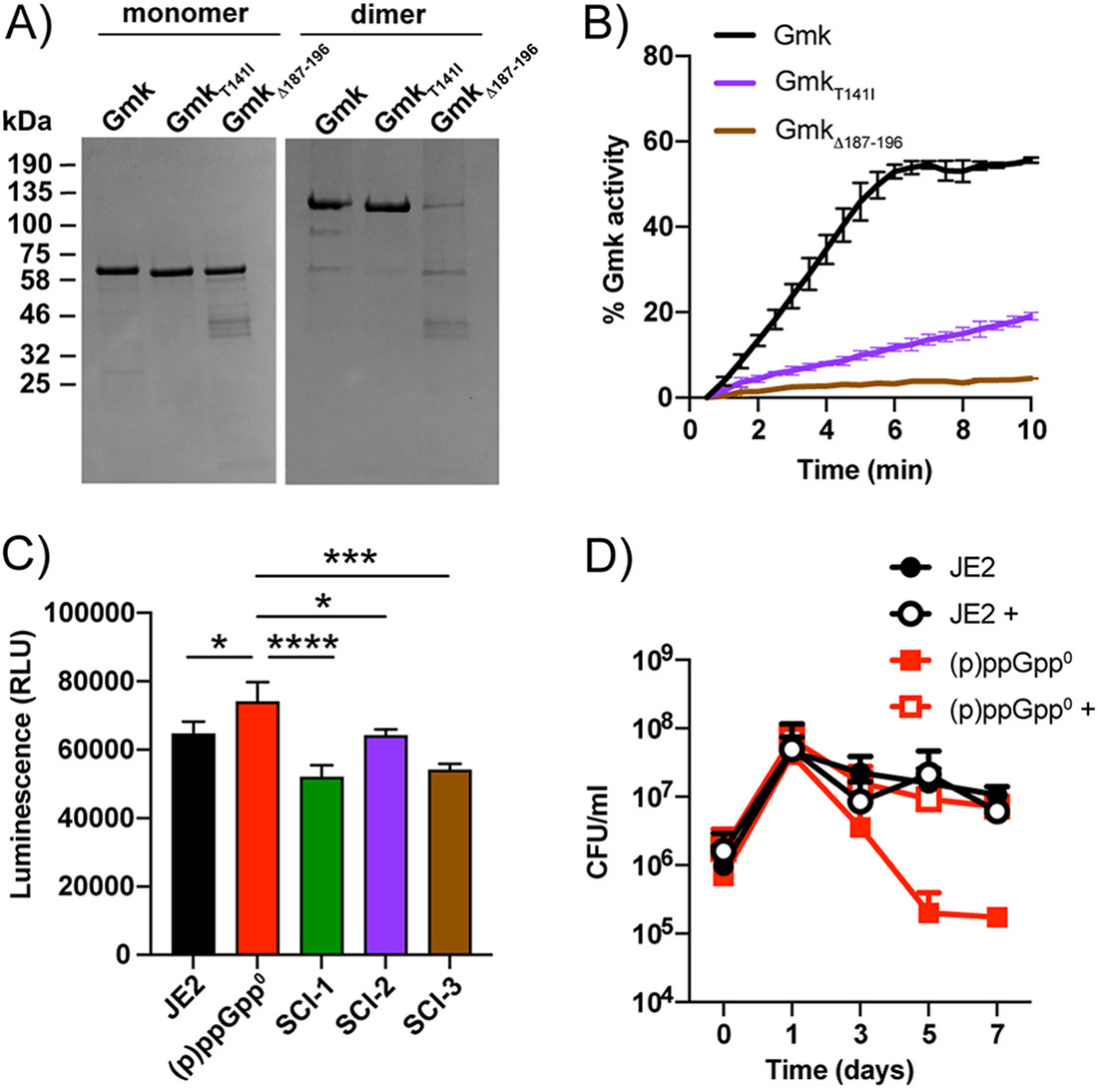
Gmk from the (p)ppGpp^0^ small colony isolates are less active than wild-type. (A) Coomassie stained gel of purified recombinant Gmk from wild-type JE2 and (p)ppGpp^0^ variants p^0^-SCI-2 and p^0^-SCI-3. Equal concentrations of Gmk, Gmk_T141I_, and Gmk_Δ187-196_ were run on 10% polyacrylamide gels under denaturing (left) or nondenaturing (right) conditions. Sizes in kDa are indicated on the left. (B) Gmk activity assay. The conversion of NADH to NAD^+^, in a Gmk-dependent manner, was monitored over time at an absorbance of 340 nm. Activity assays were performed in duplicate, with the standard deviation shown. (C) Relative cellular GTP levels. Overnight cultures of each strain were lysed and normalized by protein concentration. Relative intracellular GTP concentrations were determined via luminescence. Average luminescence values and standard deviations of triplicate experiments are plotted. Data were analyzed by one-way ANOVA with Dunnett’s multiple-comparison test, *, *P* ≤ 0.05; ***, *P* ≤ 0.001; ****, *P* ≤ 0.0001. (D) Changes in viable count (CFU/mL) were measured over 14 days for JE2 (black) and (p)ppGpp^0^ (red) in the presence (open symbols—decoyinine in DMSO) and absence (closed symbols—DMSO only) of decoyinine. Survival curves were carried out in duplicate, with error bars representing standard deviation.

To examine the impact of these mutations on the activity of Gmk, the Gmk proteins from the wild-type, p^0^-SCI-2 (Gmk_T141I_), and p^0^-SCI-3 (Gmk_Δ187-196_), which is identical to the variant from p^0^-SCI-1, were purified. When equal concentrations of the proteins were analyzed by SDS-PAGE under denaturing conditions, all three migrated at the same size of approximately 68 kDa (Fig. 5A). However, under nondenaturing conditions, Gmk_Δ187-196_ failed to efficiently form a dimer, which could be expected given the importance of helix 8 for dimer formation (42). This variant also appeared to be more susceptible to degradation. The enzymatic activity of the Gmk proteins was subsequently monitored over time with an assay that uses the conversion of GMP to GDP to ultimately measure the oxidation of NADH to NAD^+^ (19). While the wild-type enzyme was active (Fig. 5B), Gmk_T141I_ showed a marked reduction in activity, while the activity of Gmk_Δ187-196_ was not detected.

To examine the impact of these mutations on the cell, the GTP levels from the wild-type, (p)ppGpp^0^, and the three p^0^-SCIs were measured. In the absence of (p)ppGpp, cells had elevated levels of GTP (Fig. 5C), which is to be expected given the role of (p)ppGpp in regulating GTP homeostasis in B. subtilis (19, 23, 41). Compared to the (p)ppGpp^0^ parental strain, all three p^0^-SCIs had significantly reduced levels of GTP. This was more evident with p^0^-SCI-1 and p^0^-SCI-3 than with p^0^-SCI-2 (Fig. 5C), in line with the reduced but not abolished activity observed for Gmk_T141I_ (Fig. 5B). In fact, the GTP level from p^0^-SCI-2 was similar to the wild-type, which may explain why this strain had improved growth in DMEM, unlike p^0^-SCI-1 and p^0^-SCI-3 (Fig. 3A). Taken together, these data suggest that in order for S. aureus strains to survive long-term starvation in the absence of (p)ppGpp, mutations that will decrease the levels of GTP to equal or lower than wild-type levels are selected, further implicating (p)ppGpp as an important regulator of GTP homeostasis in bacteria.

### Reducing cellular GTP levels allows long-term survival of a (p)ppGpp^0^ strain.

Our data suggest that when a (p)ppGpp^0^ strain is grown in amino-acid-depleted DMEM, toxic increases in GTP levels could impede cell proliferation. To overcome this, cells with mutations that lower levels of GTP are selected, allowing proliferation, albeit more slowly than the wild-type. To test this, we repeated the long-term survival assays in DMEM but included decoyinine, an inhibitor of the GMP synthase GuaA. This inhibitor has been shown to artificially lower GTP levels and promote derepression of CodY in other organisms (43 to 45), which we hypothesize will negate the need for the (p)ppGpp^0^ strain to mutate and form small colonies. When the wild-type JE2 was grown with decoyinine, no effect on survival was observed. However, when the (p)ppGpp^0^ strain was grown in the presence of decoyinine, survival was restored to wild-type levels (Fig. 5D), and small colonies did not appear. This supports our hypothesis that during long-term starvation in amino-acid-deficient media, GTP levels increase in cells lacking (p)ppGpp, resulting in the formation of suppressor mutations. These suppressors circumvent the need for (p)ppGpp-mediated regulation of GTP homeostasis by introducing mutations in Gmk to directly reduce GTP levels and restore growth.

### The small colony phenotype of (p)ppGpp^0^ SCIs is not stable.

**FIG 6:**
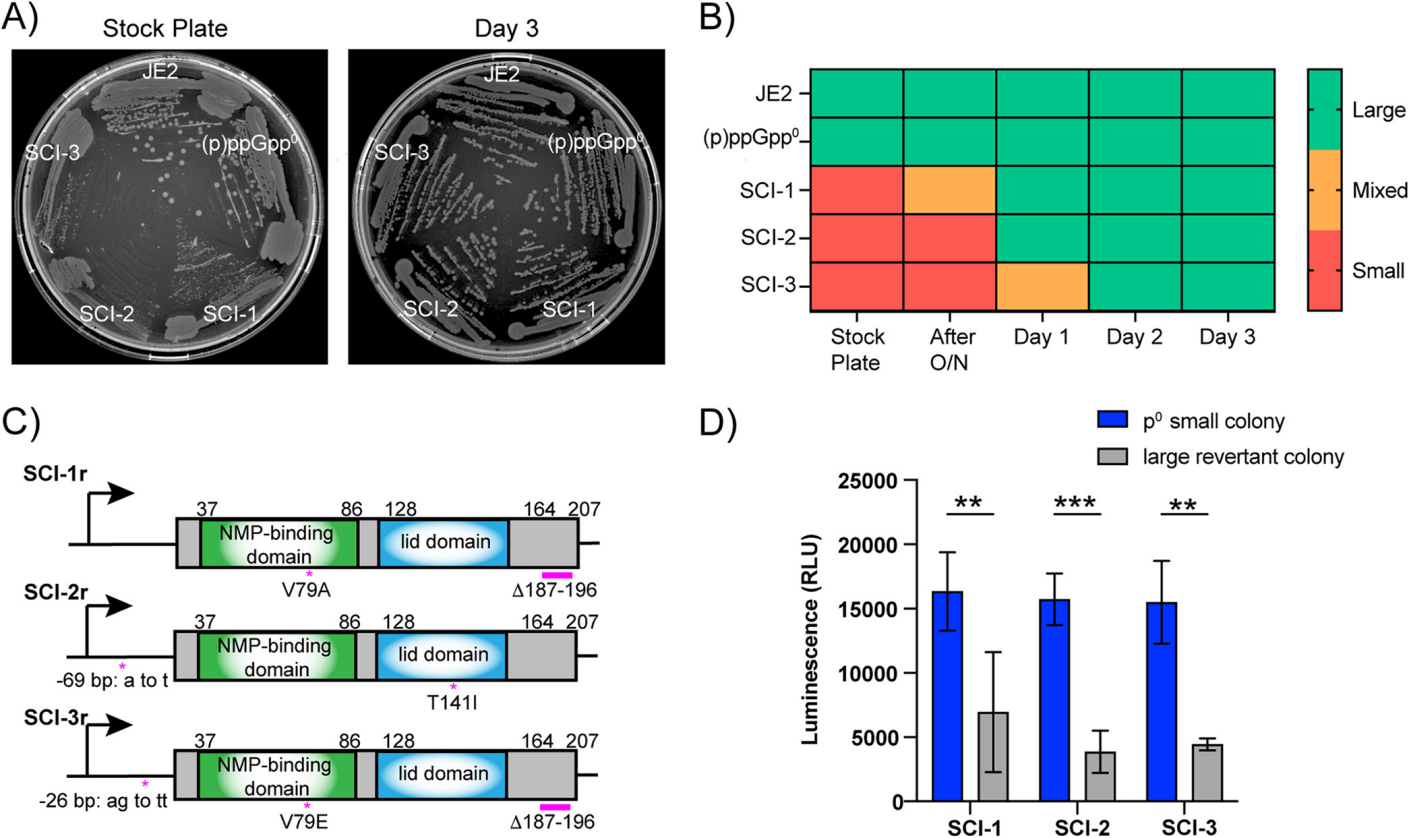
Small colony morphology of (p)ppGpp^0^ variants is not stable. (A) TSA plates showing the colony size of each strain after streaking out from frozen (stock plate) or after 3 days of subculture. (B) Color map displaying colony phenotype at each time point, starting from the original plate of frozen stocks, overnight culture, and subsequent subculture for 3 days. (C) Schematic representation of the mutations found in Gmk in the p^0^-SCI revertants. Deletions found in p^0^-SCI-1r and p^0^-SCI-3r are represented by a pink line. *, SNPs resulting in an amino acid change or changes in the upstream promoter regions. (D) Relative cellular GTP levels. Overnight cultures of each strain were lysed and normalized by protein concentration before relative GTP levels were determined by luminescence. The average luminescence values and standard deviations of triplicate experiments are plotted. Data analyzed by two-way ANOVA with Šidák’s multiple-comparison test, **, *P* ≤ 0.01; ***, *P* ≤ 0.001.

The ability of S. aureus SCVs to phenotypically switch back to a wild-type morphology under favorable conditions is a well-reported characteristic and is implicated in the ability of S. aureus to cause relapsing, chronic infections (31 to 33). To examine the phenotypic stability of the p^0^-SCIs, we plated out the strains on tryptic soy agar (TSA) after subculture for a number of days (Fig. 6A and B). All three isolates had a small colony phenotype when freshly plated out from frozen stocks (Fig. 6A). After plating an overnight culture and a number of subsequent subcultures, all three isolates had reverted to a large colony morphology similar to the wild-type and (p)ppGpp^0^ parental strains (Fig. 6A and B).

To determine if further mutations had arisen to allow the reversion to a large colony phenotype, whole-genome sequencing of three revertants, p^0^-SCI-1r, p^0^-SCI-2r and p^0^-SCI-3r, was performed. For all three, additional mutations occurred in or upstream of the *gmk* gene (Fig. 6C). Revertants p^0^-SCI-1r and p^0^-SCI-3r still contained the Δ187-196 amino acid deletion at the C terminus, but now also contained SNPs altering valine 79 to either an alanine (p^0^-SCI-1r) or a glutamic acid (p^0^-SCI-3r). V79 is within the nucleotide binding domain of Gmk (Fig. 4C) and is highly conserved across Gmk proteins within the Firmicutes (16). Furthermore, mutations were observed in the promoter region of *gmk* for both p^0^-SCI-2r and p^0^-SCI-3r, suggesting that the production of Gmk will be reduced in these revertants. This is in addition to the altered enzyme activity of any produced protein. To assess this, levels of GTP in the revertant strains were measured and compared to those of the original SCIs. All three revertants had lower levels of GTP compared to the original SCI (Fig. 6D), suggesting that further reduction of GTP levels can restore SCI colony size to wild-type. Altogether, this highlights the importance of regulating cellular GTP levels for bacterial viability.

## DISCUSSION

### Long-term survival of S. aureus in nutrient-depleted media.

In this study, we examined the requirement of a functional stringent response for long-term survival of S. aureus in nutrient-depleted media. It is well known that S. aureus can cause persistent and recurrent infections, displaying an impressive ability to survive within patients for years (36). It has been suggested that this survival could require the stringent response, as isolates from chronic infections have activated (p)ppGpp synthesis (5). This requirement may stem from host-induced nutrient restrictions, as host tissues restrict access to many metabolites, including carbon, nitrogen, metals, and amino acids (46 to 49). The evidence for restricted availability of amino acids comes from the observed upregulation of amino acid biosynthetic clusters when strains are isolated from a host (50), and the inability of numerous auxotrophic strains to cause disease (51, 52). As (p)ppGpp is essential for bacteria to respond to nutrient restrictions, this prompted our current work, where we examine the requirement of (p)ppGpp for long-term survival. By incubating wild-type and (p)ppGpp^0^ strains in nutrient-rich and nutrient-depleted media over a 2-week period, we observed survival defects for the mutant strain in both RPMI and DMEM (Fig. 1). These are tissue culture media designed to provide optimal conditions to support host tissue growth, but they differ in both iron content and amino acid composition, respectively. Previous work has suggested that (p)ppGpp is important for reducing cellular iron levels and lowering reactive oxygen species. Horvatek et al. showed that (p)ppGpp overproduction in S. aureus strains grown in chemically defined media (CDM) induced transcription of genes involved in iron storage and the oxidative stress response (30). Similarly, Fritsch et al. demonstrated that when an S. aureus (p)ppGpp^0^ strain was grown in RPMI with low iron (0.75 μM FeCl_3_), it was more susceptible to reactive oxygen species due to dysregulation of respiratory chain activity and elevated free iron in the cell (29). Here, a (p)ppGpp^0^ strain grown overnight in TSB was washed and then used to inoculate RPMI completely lacking iron. No defect in CFU was observed after 24 h (Fig. 1C), suggesting that iron levels in the RPMI were low enough to permit growth of a strain lacking (p)ppGpp in the short term. A lack of regulation in the absence of (p)ppGpp, however, may have resulted in the decreased viability observed after 7 days. Future work would be required to analyze what nutritional needs are not being met at these later time points.

Although S. aureus contains all the genes for synthesizing the 20 amino acids required for protein synthesis (53, 54), the precise conditions that might induce activity of these operons are unclear, as when cultured in the laboratory setting S. aureus is a conditional auxotroph for several amino acids, including arginine, valine, proline, cysteine, and leucine (11, 55). DMEM is deficient in a number of amino acids, including asparagine, aspartic acid, and proline, though S. aureus can synthesize proline from arginine (56). Figure 1D shows that a wild-type S. aureus strain is able to survive in DMEM over the 2-week period, suggesting that the biosynthetic operons for the missing amino acids may be switched on under this condition or that the requirement for these amino acids is very low as the cells are not actively replicating. In contrast, the (p)ppGpp^0^ strain was unable to proliferate in DMEM initially (Fig. 1D), until the formation of colonies with the smaller colony phenotype that soon dominated the population (Fig. 2). These colonies were distinct from typical SCVs, as although they were slower growing, they did not have mutations in the electron transport or thymidylate biosynthetic pathways, did not exhibit reduced hemolytic activity, and did not demonstrate reduced sensitivity to gentamicin or vancomycin (Fig. 3), all phenotypes traditionally associated with SCVs (25). Instead, these isolates contained mutations in Gmk, a key enzyme in the GTP synthesis pathway, suggesting that the regulation of GTP levels is key to the survival of a strain lacking an active stringent response in nutrient-restricted media.

### The stringent response is a regulator of cellular GTP levels.

The stringent response is most notably associated with adjusting to amino-acid-limiting environments. However, it is becoming apparent that this system is intricately linked to the regulation of cellular nucleotide levels, and has been referred to as a master regulator of GTP homeostasis under both stressed and unstressed conditions (16, 19, 23, 41). Indeed, levels of GTP and (p)ppGpp are inversely linked at multiple points. Under stringent conditions, GTP is utilized as a substrate for pppGpp synthesis. In addition, numerous studies have characterized the inhibition of multiple enzymes in both the *de novo* and salvage purine biosynthesis pathways by (p)ppGpp, including PurF, PurA, Gmk, GuaB, Gsk, HprT, XprT, and AprT (reviewed in reference 1).

In S. aureus and other Firmicutes, increased levels of (p)ppGpp and concomitant decreases in GTP relieve repression by transcriptional repressor CodY (40, 41). In S. aureus, CodY, in complex with its two cofactors GTP and branched-chain amino acids, binds to over 200 promoters, with 90% of transcripts downregulated and only 10% activated (22). Examples of downregulated transcripts include multiple amino acid biosynthetic pathways, amino acid and peptide transporters, permeases, as well as the major virulence gene regulator *agr* (22). Here, we observed that a (p)ppGpp^0^ strain has increased cellular levels of GTP (Fig. 5C, S1A) and has an initial survival defect in DMEM (Fig. 1D). The observed increase in viability in DMEM after 5 days was accompanied by mutations in Gmk that reduced its enzymatic activity (Fig. 5B), thereby reducing cellular GTP levels (Fig. 5C). Decreases in GTP have been shown to lead to the derepression of CodY and concomitant upregulation of amino acid biosynthesis in other species (43, 44), which here explains the increased viability observed for the (p)ppGpp^0^ strain that had acquired suppressor mutations at later time points (Fig. 1D). Indeed, we show that by inhibiting the uncontrolled build-up of GTP in a (p)ppGpp^0^ strain with the GuaA inhibitor decoyinine, cells no longer die and the small colony phenotype does not arise (Fig. 5D). The mutations observed in Gmk occurred in either the lid domain or within helix 8, which is crucial for dimer formation (Fig. 4). We were curious to see whether mutations in this region arise naturally or were selected for due to the absence of a functional stringent response. To this end, we performed a sequence alignment of Gmk from a collection of 990 clinical S. aureus strains isolated from lower respiratory tract infections (57). This analysis revealed that Gmk is very well conserved in S. aureus, with few mutations arising between strains and none that would appear to affect enzyme activity. This suggests that the suppressor mutations we observed are only selected for due to the toxic accumulation of GTP that arises in the absence of (p)ppGpp.

**FIG 7:**
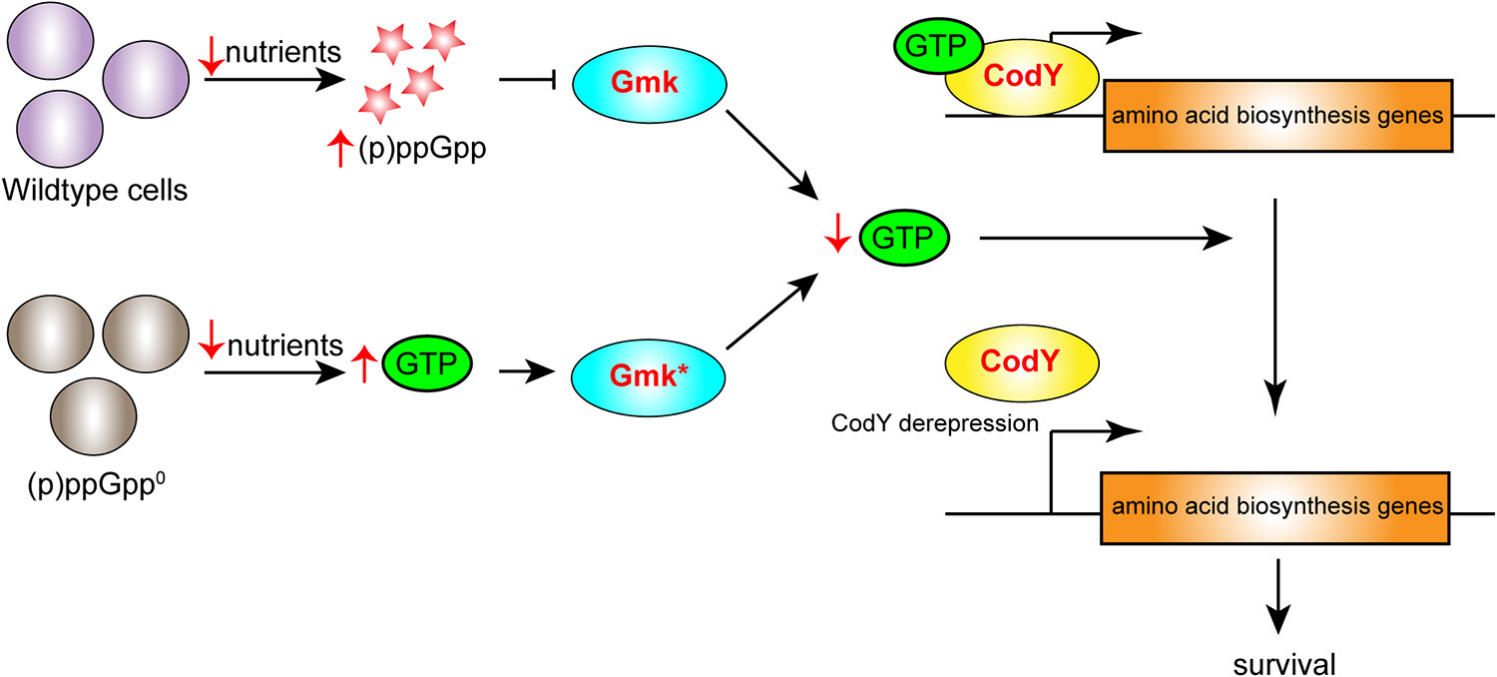
Model of the requirement of GTP for starvation survival. For wild-type S. aureus experiencing starvation, (p)ppGpp levels increase. (p)ppGpp directly binds to Gmk and a number of other enzymes in the GTP synthesis pathway, lowering intracellular levels of GTP. GTP is a cofactor required for the activity of transcriptional repressor CodY. When GTP levels are lowered, CodY is derepressed, leading to the transcription of amino acid biosynthesis genes and survival. In the absence of (p)ppGpp, GTP levels rise when cells are starved as there is no inhibition of GTP synthesis enzymes. The bacteria respond by introducing mutations in Gmk (denoted as Gmk*) that lower activity, resulting in lower levels of GTP and survival.

From these results, we propose a model that, in line with observations in other species (19), highlights that a major function for (p)ppGpp in cells is the moderation of GTP levels (Fig. 7). When nutrients are limited, cells produce (p)ppGpp, which will inhibit GTP production by the inhibition of several enzymes in the synthesis pathway, including Gmk. In the Firmicutes, lowered GTP levels will induce derepression of CodY and increase amino acid biosynthesis. Similar to these observations, in B. subtilis GTP levels reportedly rise uncontrollably in cells that lack (p)ppGpp, leading to toxicity and cell death within a few hours (19, 23). GTP is involved in multiple cellular processes in addition to the regulation of CodY activity, including transcription and ribosome biogenesis, and as such, dysregulation could have pleiotropic detrimental impacts on cell survival. However, our long-term viability study shows that bacteria can introduce suppressor mutations to lower the toxic levels of GTP, alleviating the need for (p)ppGpp-mediated regulation.

Altogether, this work has shown that, similar to B. subtilis, (p)ppGpp is crucial for regulating cellular GTP levels in S. aureus. Cells lacking (p)ppGpp readily acquire suppressor mutations, allowing for restoration of GTP homeostasis and cell survival. The ability of bacteria to overcome the need for this signaling system would call into question attempts to target (p)ppGpp as a therapeutic strategy.

## MATERIALS AND METHODS

### Bacterial strains and culture conditions.

E. coli strains were grown in Luria-Bertani broth (LB). S. aureus strains were grown in tryptic soy broth (TSB; Becton, Dickinson); Roswell Park Memorial Institute (RPMI) 1640 medium (Gibco 22400089); low-glucose Dulbecco’s Modified Eagle’s Medium (DMEM; Merck D6046) supplemented with 2 mM l-glutamine and 10% FBS; or human serum from male AB plasma (Merck H4522) at 37°C.

### Plasmid and strain construction.

Strains used in this study are listed in Table S2, and primers used are listed in Table S3. Plasmids pVL847-*gmk_SCI-2_* and pVL847-*gmk_SCI-3_* were produced by amplifying the respective *gmk* genes using primers RMC920/RMC921. The resulting PCR products were digested with BamHI and EcoRI and cloned into pVL847 that had been digested with the same enzymes. All plasmids were initially transformed into E. coli strain XL1-Blue, and the sequences of all the inserts were verified by fluorescence automated sequencing by Eurofins. pVL847 plasmids were subsequently transformed into BL21(DE3) for protein induction.

For deletion of the *relP*, *relQ,* and *rel* genes in S. aureus, 1-kb fragments up- and downstream of each gene were amplified from JE2 genomic DNA using primer pairs as specified in the primer table. For Rel, this results in a 234 amino acid in-frame deletion spanning the hydrolase and synthetase domains. For RelP and RelQ, both in-frame deletions lacked 29 amino acids containing the syn3 and syn4 synthetase motifs. Purified PCR products were then fused by splice overlap extension (SOE) PCR using flanking primer pairs. The purified PCR product was cloned into pIMAY and transformed into E. coli strain XL1-Blue. The plasmid was subsequently electroporated into RN4220 and stably maintained at 30°C in the presence of 10 μg/mL chloramphenicol (Cam). Electroporation of the plasmids into JE2 then allowed mutant construction as previously outlined (58). Replacement of each gene was confirmed by PCR, and whole-genome sequencing was performed on the final triple (p)ppGpp^0^ strain, to ensure the absence of secondary mutations. Genomic DNA extraction and whole-genome sequencing were performed by MicrobesNG (Birmingham, UK).

### Starvation assay.

S. aureus strains were cultured overnight in 5 mL TSB at 37°C with shaking. Cultures were washed three times with PBS, resuspended in the appropriate media, and used to inoculate 10 mL of either TSB, human serum, RPMI, or DMEM to an OD_600_ of 0.01. Cells were incubated with shaking at 37°C for 24 h (day 1), followed by static incubation until day 14. CFU from days 0, 1, 3, 5, 7, and 14 were enumerated on TSA from plates incubated for 24 h at 37°C. Colony size was determined using the analyze particle tool in ImageJ. Where stated, 50 μg/mL decoyinine solubilized in DMSO, or a DMSO-only control, were included at the time of inoculation.

### Growth curves.

S. aureus strains were cultured overnight in 5 mL TSB at 37°C with shaking. Strains were subsequently normalized to OD_600_ of 0.05 in TSB plus or minus 0.05 μg/mL mupirocin as stated in the legend. For growth curves in DMEM, the cultures were washed three times with DMEM before normalization. The OD_600_ was measured every 15 min for 15 h in a Hidex Sense microplate reader.

### MICs.

Overnight cultures of S. aureus strains were adjusted to an OD_600_ of 0.05 in Mueller-Hinton broth and 100 μL incubated in 96-well plates with 2-fold dilutions of various antimicrobials at the following starting concentrations: mupirocin 1 μg/mL, vancomycin 32 μg/mL, and gentamicin 32 μg/mL. Plates were incubated at 37°C overnight with shaking and the OD_600_ determined using a Hidex Sense microplate reader.

### Protein purification.

Proteins were purified from 500 mL E. coli BL21 DE3 cultures. Cultures were grown at 37°C to an OD_600_ of 0.5 to 0.7; expression was induced with 1 mM isopropyl β-D-1-thiogalactopyranoside (IPTG) and incubated for 3 h at 30°C. Cell pellets were resuspended in 5 mL Buffer A (50 mM Tris pH 7.5, 150 mM NaCl, 5% glycerol, 10 mM imidazole) and lysed by sonication upon addition of 20 μg/mL lysozyme. Protein purifications were performed by nickel affinity chromatography. The filtered cell lysate was loaded onto a 1-mL HisTrap HP Ni^2+^ column (GE Healthcare) before elution using a gradient of Buffer B (50 mM Tris pH 7.5, 200 mM NaCl, 5% glycerol, 500 mM imidazole). Protein-containing fractions were dialyzed in 50 mM Tris-HCl pH 7.5, 200 mM NaCl, 5% glycerol before concentrating using a 50-kDa centrifugal filter (Thermo Scientific) and storage at −80°C. For analysis of dimer formation, samples were mixed with a loading dye lacking β-mercaptoethanol and were not heated prior to analysis on SDS-PAGE. For use in Gmk enzymatic assays, proteins were further purified by size exclusion chromatography using a preparative 16/60 Superdex 200 column and a 50 mM Tris pH 7.5, 200 mM NaCl, 5% glycerol buffer system.

### Measurement of (p)ppGpp levels in S. aureus.

S. aureus strains were grown overnight in low-phosphate CDM (59) at 37°C. Cultures were diluted to an OD_600_ of 0.05 and grown until an OD_600_ of 0.5. Following this, 3.7 MBq of [^32^P]H_3_PO_4_ was added to 500 μL cultures and incubated for a further 3 h at 37°C. Cultures were subsequently normalized for absorbance, supplemented with 60 μg/mL mupirocin, and further incubated for 30 min at 37°C. Cultures were pelleted and suspended in 100 μL of 600 mM formic acid, before being subjected to three freeze/thaw cycles. Samples were centrifuged at 17,000 × *g* for 5 min. Ten microliters of the supernatant fractions were subsequently spotted on PEI-cellulose F thin-layer chromatography (TLC) plates (Merck Millipore), nucleotides separated, and TLC plates developed using a 1.5 M KH_2_PO_4_ pH 3.6 buffer. The radioactive spots were visualized using an FLA 7000 Typhoon PhosphorImager, and data were quantified using ImageQuantTL software.

### Enzymatic assays.

The Gmk activity assay contained 100 mM Tris HCl pH 7.5, 100 mM KCl, 10 mM MgCl_2_, 4 mM ATP, 1.5 mM phosphoenolpyruvate, 2 U of pyruvate kinase, 2.64 U of lactate dehydrogenase, 150 μM NADH, and 10 μM Gmk variants. Reactions were initiated by the addition of 1.25 mM GMP, and the absorbance at 340 nm was monitored over time.

### Relative GTP concentration determination.

A GTPase-Glo bioluminescence assay (Promega) was used, and manufacturer’s guidelines were adjusted to measure relative intracellular GTP levels by luminescence. Bacterial cultures were pelleted and resuspended in TSM buffer (50 mM Tris pH 7.5, 0.5 M sucrose, 10 mM MgCl_2_) before lysing with 50 μg/mL lysostaphin and 50 μg/mL DNase for 30 min at 37°C. Total protein concentrations were determined using a Bicinchoninic Acid (BCA) assay (Bio-Rad). The protein concentration of each lysate was adjusted to 100 μg/mL and 2.5 μL (250 ng) used in each GTPase-Glo assay.

### Stability assay.

S. aureus strains were plated onto TSA from frozen stocks (stock plate). Single colonies were inoculated in 5 mL TSB and incubated overnight at 37°C with shaking. Overnight cultures were plated onto TSA to examine morphology, and 1:1,000 dilutions were used to inoculate day 1 cultures. Day 1 cultures were incubated for 24 h at 37°C, with shaking. The following day, aliquots were plated onto TSA and day 2 cultures prepared by inoculating 5 μL into 5 mL fresh TSB. The aforementioned steps were repeated until day 3. Colonies were stocked from TSA plates at each time point, and overnight cultures were used to prepare protein lysates and isolate chromosomal DNA for sequencing.

### Statistics.

Statistical analyses were performed using GraphPad Prism 8.0 software. Statistical differences between samples were assessed using either two-tailed, unpaired *t* testing or one- or two-way analysis of variance (ANOVA), followed by multiple-comparison test, as indicated in the figure legends.

### Data availability.

Whole-genome sequence data are available from the European Nucleotide Archive (Study Accession number PRJEB58759), accession numbers ERS14397334 to ERS14397338. The SAUSA300_FRP3757 (TaxID: 451515) reference genome sequence is available from NCBI, while the clinical isolate sequences used to look at Gmk protein sequences in wild-type strains are available at PRJEB2756.
