# Supplementary material for: Stringent Response-Mediated Control of GTP Homeostasis Is Required for Long-Term Viability of Staphylococcus aureus
Source: Microbiol Spectr. 2023 Mar 6;11(2):e00447-23. doi: 10.1128/spectrum.00447-23 (PMC10101089; doi:10.1128/spectrum.00447-23)
Supplement: Supplemental file 1 — Supplemental material. Download spectrum.00447-23-s0001.docx, DOCX file, 10.6 MB [file spectrum.00447-23-s0001.docx]

**SUPPLEMENTAL**

**
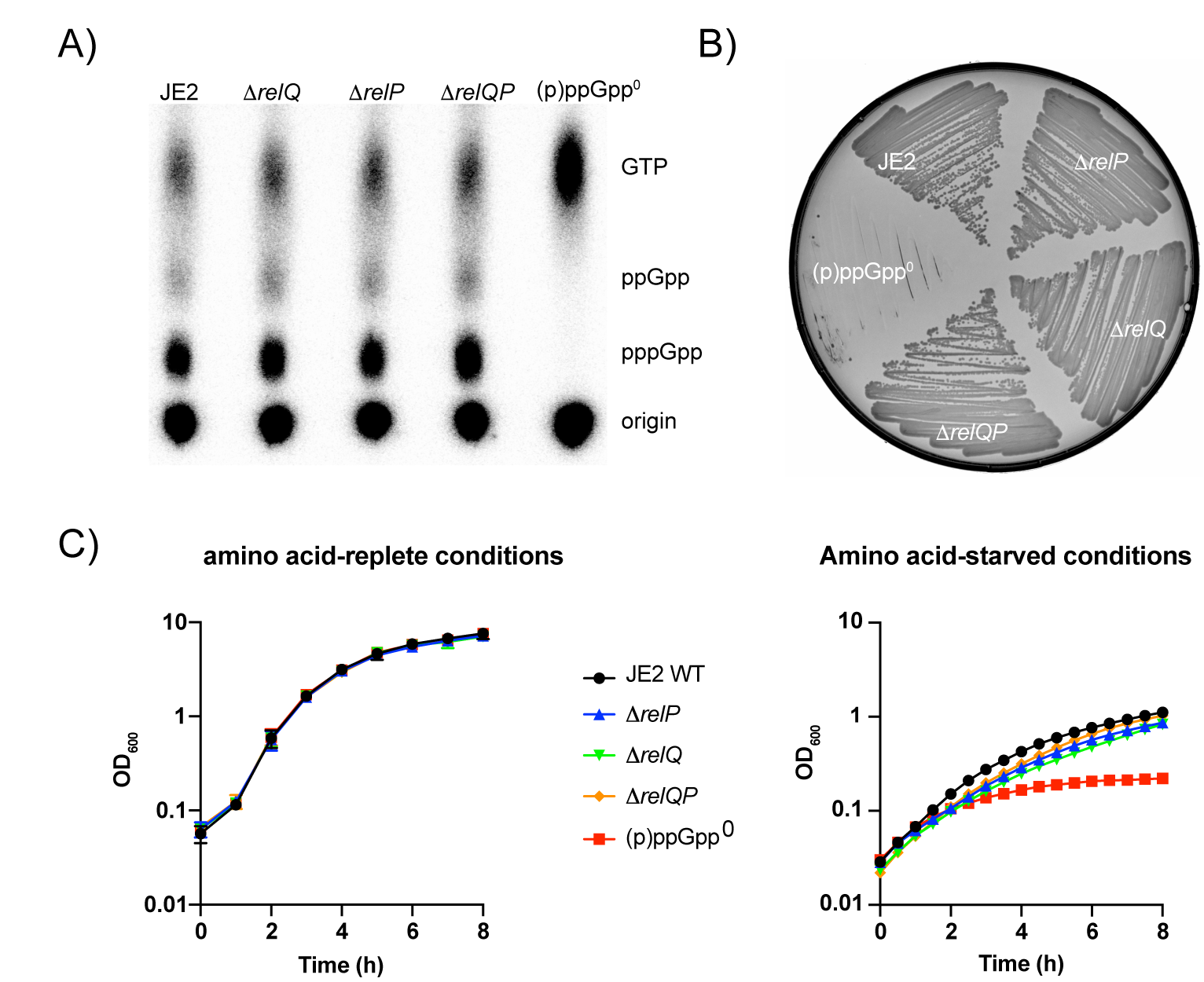
**

**FIG S1 Characterisation of (p)ppGpp^0^ *S. aureus* mutant strains.** A) Measurement of intracellular (p)ppGpp levels in single, double and triple (p)ppGpp synthetase mutant strains. Bacteria were grown in the presence of ^32^P-H_3_PO_4_ and the stringent response was induced by the addition of mupirocin for 30 min. The production of pppGpp and ppGpp was monitored by TLC. Radiolabelled GTP, pppGpp and ppGpp produced *in vitro* were run in parallel to identify the relevant spots. B) Mutant strains were streaked on TSA agar plate containing 0.05 μg/ml mupirocin, confirming that the triple mutant cannot survive amino acid starvation without (p)ppGpp. C) Growth curves monitoring the growth of (p)ppGpp-synthetase mutant strains in amino acid-replete (TSB) or amino acid-starved (plus 0.05 μg/ml mupirocin) conditions. Overnight cultures were diluted to an OD_600_ of 0.05 and grown for 8 hr. Graphs show the mean OD_600_ of three technical replicates, with the standard deviation.

**
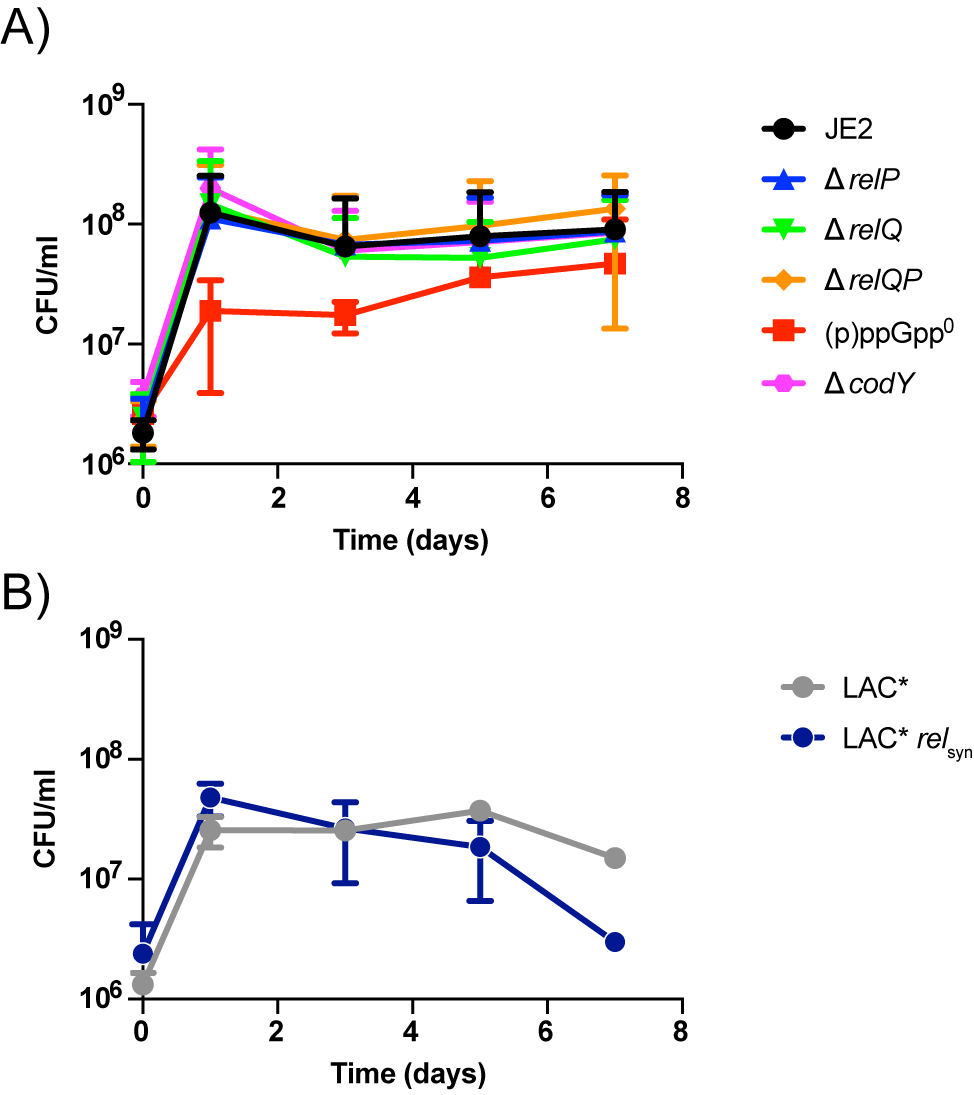
**

**FIG S2 Survival of wildtype and (p)ppGpp-synthetase single, double and triple mutant strains in DMEM.** Changes in viable count (CFU/ml) were measured over 14 days for: A) the wildtype JE2, the single (p)ppGpp mutants Δ*relP* and Δ*relQ*, the double mutant Δ*relQP*, the (p)ppGpp^0^ strain and a Δ*codY* mutant; and B) for the USA300 strain LAC* and a Rel synthetase mutant incapable of producing (p)ppGpp. Survival curves were carried out in duplicate, with error bars representing standard deviation.

**TABLE S1 Growth rate measurements**

| **Strain** | **Maximum Growth Rate (ΔOD_600_/hr)**  **TSB** | **Relative to (p)ppGpp^0^**  **TSB** | **Maximum Growth Rate**  **(ΔOD_600_/hr)**  **DMEM** |  | **Relative to (p)ppGpp^0^**  **DMEM** |
| --- | --- | --- | --- | --- | --- |
| JE2 | 0.370 ± 0.020 | 0.979 | 0.112 ± 0.008 |  | 1.188 |
| (p)ppGpp^0^ | 0.378 ± 0.026 | 1 | 0.094 ± 0.009 |  | 1 |
| p^0^-SCI-1 | 0.265 ± 0.035 | 0.702 | 0.076 ± 0.012 |  | 0.807 |
| p^0^-SCI-2 | 0.245 ± 0.015 | 0.650 | 0.120 ± 0.008 |  | 1.268 |
| p^0^-SCI-3 | 0.199 ± 0.012 | 0.526 | 0.074 ± 0.038 |  | 0.787 |

**TABLE S2 Bacterial strains used in this study**

| **Strain** | **Relevant features** | **Reference** |
| --- | --- | --- |
|  | ***Escherichia coli* strains** |  |
| XL1-Blue | Cloning strain: TetR | Stratagene |
| BL21 (DE3) | Strain used for protein expression | Novagen |
| pVL847 | His-MBP fusion vector: CarbR | (1) |
| pVL847-*gmk* | His-MBP-Gmk: CarbR | (2) |
| pVL847-*gmk* 2.1 | His-MBP-Gmk_T141I_: CarbR | This study |
| pVL847-*gmk* 3.1 | His-MBP-Gmk_Δ187-196_: CarbR | This study |
|  |  |  |
|  | ***Staphylococcus aureus* strains** |  |
| JE2 | CA-MRSA USA300 strain LAC derivative, lacking plasmids p01 and p03. Erm sensitive | (3) |
| JE2 Δ*relP* | JE2 with in-frame deletions in *relP* | This study |
| JE2 Δ*relQ* | JE2 with in-frame deletions in *relQ* | This study |
| JE2 Δ*relQP* | JE2 with in-frame deletions in *relQ* and *relP* | This study |
| JE2 Δ*relQPA* | JE2 with in-frame deletions in *relQ*, *relP* and *rel*: ((p)ppGpp^0^) | This study |
| JE2 Δ*codY* | NE1555. JE2 with transposon insertion in *codY*: ErmR | (3) |
| LAC* | Erm sensitive CA-MRSA LAC strain (AH1263) | (4) |
| LAC *rel*_syn_ | Deleted nt 405-536 | (5) |
| p^0^-SCI-1 | JE2 Δ*relQPA* with deletion in *gmk* | This study |
| p^0^-SCI-2 | JE2 Δ*relQPA* with mutation in *gmk* | This study |
| p^0^-SCI-3 | JE2 Δ*relQPA* with deletion in *gmk* | This study |
| p^0^-SCI-1-r | JE2 Δ*relQPA* with mutation and deletion in *gmk* | This study |
| p^0^-SCI-2-r | JE2 Δ*relQPA* with mutations in *gmk* | This study |
| p^0^-SCI-3-r | JE2 Δ*relQPA* with mutation and deletion in *gmk* | This study |

Antibiotics were used at the following concentrations - for *E. coli* cultures: carbenicillin (CarbR) 50-150 μg/ml. IPTG was used at 1 mM. For *S. aureus* cultures: erythromycin 10 μg/ml **.**

**TABLE S3 Primers used in this study**

| **Number** | **Name** | | **Sequence** |
| --- | --- | --- | --- |
| RMC920 | F-NdeI-Gmk | gggcatatgGATAATGAAAAAGGATTGTTAATCGTTTTATC | |
| RMC921 | R-BamHI-Gmk | gggggatccTTATTTTTTAGCCTCCAAAATCATTTTTC | |
| RMC927 | F-KpnI-Rel-up | gggGGTACCgcaattaaaccaggtcaacgtg | |
| RMC928 | R-Rel-up | tgtagtatgcaacaaaaaacctgcgacaatcgtcgg | |
| RMC929 | F-Rel-down | gcaggttttttgttgcatactacagtagtaggccca | |
| RMC930 | R-SacI-Rel-down | gggGAGCTCgggcaatcagtgcgatgtac | |
| RMC931 | F-KpnI-RelP-up | gggGGTACCcaaagcattagaagaagagaac | |
| RMC932 | R-RelP-up | ATCCATACCTATACCATTTTCTTTAGGGTGCTGAAT | |
| RMC933 | F-RelP-down | AAAGAAAATGGTATAGGTATGGATATGTGGGCAAGT | |
| RMC934 | R-SacI-RelP-down | gggGAGCTCctggcagttaggacataaatagg | |
| RMC935 | F-KpnI-RelQ-up | gggGGTACCgatatgtatacacctcgtatcaaattc | |
| RMC936 | R-RelQ-up | ATTCATTGCTAAACCACTTTCTTTAGTGTTACGAAT | |
| RMC937 | F-RelQ-down | AAAGAAAGTGGTTTAGCAATGAATTTCTGGGCAACG | |
| RMC938 | R-SacI-RelQ-down | gggGAGCTCcatttgctacacggtattgtattg | |

Restriction sites in primer sequences are underlined

**References**

1. Roelofs KG, Jones CJ, Helman SR, Shang X, Orr MW, Goodson JR, Galperin MY, Yildiz FH, Lee VT. 2015. Systematic Identification of Cyclic-di-GMP Binding Proteins in *Vibrio cholerae* Reveals a Novel Class of Cyclic-di-GMP-Binding ATPases Associated with Type II Secretion Systems. PLoS Pathog 11:e1005232. doi:10.1371/journal.ppat.1005232.
2. Corrigan RM, Bellows LE, Wood A, Grundling A. 2016. ppGpp negatively impacts ribosome assembly affecting growth and antimicrobial tolerance in Gram-positive bacteria. Proc Natl Acad Sci U S A 113:E1710-9. doi:10.1073/pnas.1522179113.
3. Fey PD, Endres JL, Yajjala VK, Widhelm TJ, Boissy RJ, Bose JL, Bayles KW. 2013. A genetic resource for rapid and comprehensive phenotype screening of nonessential *Staphylococcus aureus* genes. mBio 4:e00537-12. doi:10.1128/mBio.00537-12.
4. Boles BR, Thoendel M, Roth AJ, Horswill AR. 2010. Identification of genes involved in polysaccharide-independent *Staphylococcus aureus* biofilm formation. PLoS One 5:e10146. doi:10.1371/journal.pone.0010146.
5. Corrigan RM, Bowman L, Willis AR, Kaever V, Grundling A. 2015. Cross-talk between two nucleotide-signaling pathways in *Staphylococcus aureus*. J Biol Chem 290:5826-5839. doi:10.1074/jbc.M114.598300.
